# The Role of Neutrophil-to-Lymphocyte Ratio and Platelet-to-Lymphocyte Ratio in Predicting Atrial Fibrillation and Its Comorbidities

**DOI:** 10.3390/life15060960

**Published:** 2025-06-16

**Authors:** Evelina Maria Gosav, Daniela Maria Tanase, Anca Ouatu, Oana Nicoleta Buliga-Finis, Diana Popescu, Cristina Gena Dascalu, Nicoleta Dima, Minerva Codruta Badescu, Ciprian Rezus

**Affiliations:** 1 Department of Internal Medicine, “Grigore T. Popa” University of Medicine and Pharmacy, 700115 Iasi, Romania; dr.evelinagosav@gmail.com (E.M.G.); ank_mihailescu@yahoo.com (A.O.); oana_finish@yahoo.com (O.N.B.-F.); dr.popescu.diana@gmail.com (D.P.); nicoleta.dima@umfiasi.ro (N.D.); minerva.badescu@umfiasi.ro (M.C.B.); ciprianrezus@yahoo.com (C.R.); 2 Internal Medicine Clinic, “St. Spiridon” County Clinical Emergency Hospital, 700111 Iasi, Romania; 3 Department of Medical Informatics and Biostatistics, Faculty of Medicine, “Grigore T. Popa” University of Medicine and Pharmacy, 16 University Street, 700115 Iasi, Romania; cristina.dascalu@umfiasi.ro

**Keywords:** atrial fibrillation, AF, neutrophil-to-lymphocyte ratio, NLR, platelet-to-lymphocyte ratio, PLR, chronic kidney disease, CKD, type 2 diabetes mellitus, T2DM

## Abstract

Atrial fibrillation (AF) is one of the most common cardiac arrhythmias encountered globally, characterized by a pro-inflammatory pattern. This analysis evaluated the neutrophil-to-lymphocyte ratio (NLR) and platelet-to-lymphocyte ratio (PLR) in patients with AF alongside chronic kidney disease (CKD) and/or type 2 diabetes mellitus (T2DM). This retrospective cohort study included 6077 patients admitted to the Third Medical Clinic of Saint Spiridon Hospital in Iasi from 2018 to 2023, all diagnosed with AF, CKD, and T2DM. After applying the exclusion criteria, 1066 AF patients remained eligible. For a multivariate comparative analysis, the patients were divided into groups: I. control group (non-AF patients); II. AF patients; III. T2DM group; IV. CKD-only group; V. AF+CKD group; VI. AF+T2DM group; and VII. AF+T2DM+CKD group. The Mann–Whitney/Kruskal–Wallis test demonstrated a statistically significant difference in NLR and PLR values between the AF group and the non-AF group (H = 70.627, *p* < 0.001). The receiver operating characteristic (ROC) analysis identified statistical significance and predictive power for NLR (AUC = 0.722; sensitivity = 63.6%; specificity = 76.9%) and neutrophil count in diagnosing AF, T2DM, and CKD. In conclusion, this study illustrated the utility of NLR and PLR as readily available and predictive biomarkers of inflammation in patients with AF, with or without comorbidities.

## 1. Introduction

Atrial fibrillation (AF) remains the most frequent sustained arrhythmia globally, with an increasing incidence and prevalence that subsequently impairs quality of life. The European Society of Cardiology (ESC) and the American College of Cardiology/American Heart Association (AHA) have introduced new guidelines that emphasize improved therapeutic options and the significance of risk assessment and AF prevention. In most cases, AF patients present with multiple comorbidities, both cardiovascular (CVD) (e.g., arterial hypertension, heart failure) and non-CVD (e.g., type 2 diabetes mellitus (T2DM), chronic kidney disease (CKD), metabolic syndrome, sleep apnea syndrome), which affect the onset, progression, prognosis of AF, and the therapeutic approach [1,2]. 

Although new CVD risk assessment tools and novel predictive biomarkers, such as long non-coding RNAs (lncRNAs), are under research, some analyses are far from being implemented in clinical practice due to high costs [3]. Common inflammatory patterns and oxidative stress characterize cardiac disease and other non-inflammatory disorders; therefore, white blood cells (WBCs) and their progenitors, specifically circulating leukocyte-based indices, have emerged as novel potential cardiac pro-inflammatory predictive or prognostic biomarkers [4,5]. The neutrophil-to-lymphocyte ratio (NLR) and platelet-to-lymphocyte ratio (PLR) are two easily obtainable inflammatory biomarkers that have shown predictive and valuable prognostic roles in CVD conditions such as AF [6,7], heart failure [8], obesity and metabolic syndrome [9], diabetes [10], and chronic kidney disease [11]. These two ratios indicate systemic inflammation and reflect enhanced immune system activity. If neutrophils and platelets display ongoing inflammation, lymphocytes serve as markers of regulatory pathways, with a low count indicating physiological stress. 

Given their heterogeneous pattern, establishing specific cut-off values for each disease is challenging. The current literature indicates that some studies have examined the role of NLR and PLR in AF, highlighting their superior potential in combination as early predictors of both short- and long-term outcomes in AF patients [12]. However, few have explored their relationship in patients with AF and other comorbidities. Therefore, this study aims to evaluate the utility of these two readily available inflammatory biomarkers (NLR and PLR) in a multivariate comparative analysis in patients with AF, with or without comorbidities such as T2DM and CKD.

## 2. Materials and Methods

### 2.1. Study Design and Extraction Data

We conducted a retrospective cohort unicentric study that included 6077 patients admitted to the Third Medical Clinic of the Saint Spiridon Emergency Hospital in Iasi, Romania, over 5 years (December 2018–December 2023). Diagnoses were obtained using ICD-10 codes and retrieved from InfoWorld, an electronic medical database for shared comprehensive patient records across public hospitals and clinics in our country. AF was defined as a previously documented diagnosis and current antiarrhythmic treatment, or newly diagnosed AF identified via electrocardiogram in 12 derivations [1]. CKD diagnosis was asserted via Kidney Disease: Improving Global Outcomes (KIDGO) Guidelines definition as abnormalities of kidney structure or function, present for a minimum of three months, with health implications, prior CKD diagnosis or newly found CKD based on GFR (mL/min per 1.73 m^2^) and additional laboratory (urinalysis and urine sediment) and imaging tests (ultrasound) [13]. Diabetes was defined as fasting plasma glucose (FPG) ≥ 126 mg/dL (≥7.0 mmol/L) on two separate occasions or a value for glycosylated hemoglobin 6.5% according to American Diabetes Association (ADA) guidelines or current antidiabetic treatment and documented disease [14].

**Figure 1:**
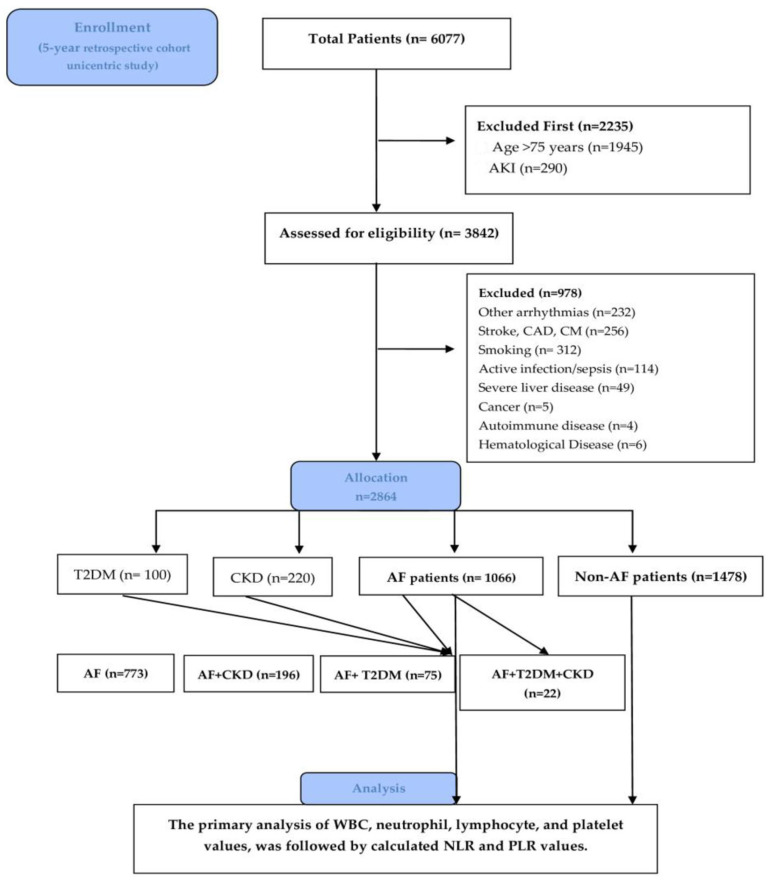
Consort flow diagram of the patients included. Cardiomyopathy (CM); coronary artery disease (CAD); acute kidney injury (AKI); atrial fibrillation (AF); type 2 diabetes mellitus (T2DM); chronic kidney disease (CKD); white blood cell (WBC).

Patients who fulfilled the following criteria were included: patients aged between 18 and 75 years, diagnosed with AF, T2DM, and CKD. Due to their heterogeneity, as their values rise independently with age, particularly NLR [15], we excluded patients over 75 years old. The other exclusion criteria were as follows: other arrhythmias (such as atrial flutter or ventricular arrhythmia); acute kidney disease (AKI); primary factors for acute or chronic inflammation: active smoking, infections (like sepsis), and hematologic–oncological diseases (cancer and leukemia); severe liver disease; autoimmune diseases (systemic lupus erythematosus, rheumatoid arthritis); patients on steroid medication; pregnancy (n = 0). Additional comorbidities such as obesity were also documented. The flow chart diagram in Figure 1 displays the study design, process, and number of patients selected.

Baseline laboratory data for complete blood count (lymphocyte, neutrophil, platelet count, and hemoglobin level), renal function tests (creatinine, sodium, potassium, and urea), lipid profiles (high-density lipoprotein cholesterol [HDL-C], low-density lipoprotein cholesterol [LDL-C], total cholesterol, and triglycerides), and glycemic profile (HbA1c) were also collected at admission. NLR was calculated as absolute neutrophil count, cells/μL)/absolute lymphocyte count, cells/μL. PLR was also calculated using the absolute platelets and lymphocyte values from the complete blood count.

### 2.2. Statistical Analysis

Data analysis was performed using SPSS 29.0. Frequencies, averages, and standard deviations characterized the variability analysis presented. The comparison between samples was achieved using the Mann–Whitney U test and Kruskal–Wallis nonparametric tests; logistic regression analysis and receiver operating characteristic curve (ROC) analysis were used for statistical analysis. Previously, via the Kolmogorov–Smirnov Fit test, the law of repartition (equality of continuous one-dimensional probability distributions) was checked for not respecting the quantitative variables. It identified significant statistical differences in patients with and without arrhythmia–AF. The cut-off value of NLR and PLR and each parameter was determined using the ROC analysis. Values < 0.05 were considered significative statistics, and a value of *p* < 0.01 was considered high significative statistics.

### 2.3. Ethics Statement

This study was approved by the Institutional Review Board (IRB) and Ethics Committee of the Third Medical Clinic of the Saint Spiridon Hospital in Iasi (number Nr. 113/20.12.2022) and by The University of Medicine and Pharmacy “Grigore T. Popa” Iasi Research Ethics Committee (number Nr. 302/16.05.2023). Due to the study characteristics (retrospective analysis), patient consent was waived.

## 3. Results

### 3.1. Baseline Characteristic

After the exclusion criteria were applied, 1084 subjects with AF remained for the study and 1066 patients to whom the complete blood count was applied. For a multivariate comparative analysis, the patients were divided into groups: I. control group non-AF patients (n = 1478, 51%); II. AF-patients (n = 1066, 36.9%); III. T2DM-only group (n = 100, 3.4%); IV. CKD-only group (n = 220, 7.5%); V. AF+CKD group (n = 197, 6.7%); VI. AF+T2DM (n = 75, 2.6%); and VII. AF+T2DM+CKD group (n = 22, 0.7%). The biological parameters and demographic characteristics of the population included are summarized in Table 1 and the Supplementary Materials.

There were significant statistical differences between genders regarding AF and comorbidities (Pearson Chi-squared test Chi2 = 27.092, *p* < 0.001). The share of women without pathology is significantly higher than the share of men (n = 576, 54.4% compared to n = 508, 48.2%); on the other hand, the percentage of AF is higher in men (30.4%) compared to women (23.8%), while the shares of AF accompanied by the two comorbidities, T2DM and CKD, are relatively low in both sexes (11.0% in men and 9.1% in women), Supplementary Materials (Tables S1 and S2, Figure S1).

Statistically significant differences were also observed between age groups (Chi2 = 176.376, *p* < 0.001), Supplementary Materials (Figure S2, Table S3). Thus, in the age group under 40 years, 89.9% of patients have no pathology, and the share of AF is only 7.9%. In the 40–60 years age group, the share of patients without pathology decreases to 67.2% and AF cases reach 21.3%, with a low percentage of 3.4% of AF accompanied by T2DM and/or CKD. In patients over 60 years, cases without pathology decrease to 45.2%, while 29.4% of patients present AF and 12.5% present AF associated with T2DM and CKD.

Additionally, according to the origin, there is a significant statistical difference in subjects from rural vs. urban areas (Chi2 = 21.961, *p* = 0.003). In this case, the urban patients display a healthier pattern compared to subjects from rural areas (non-AF 55.4% vs. non-AF 8.4%); 29.8% of cases with AF were recorded in rural patients compared to 23.3% in the urban area, and 10.7% of AF+T2DM+CKD cases in rural areas compared to 9.2% in urban areas, Supplementary Materials (Figure S3, Table S4).

### 3.2. Association Between AF, Obesity, and Dyslipidemia

Among the evaluated groups, patients with obesity and dyslipidemia were also assessed between non-AF and AF groups. In the group of patients with dyslipidemia, a statistically significantly higher proportion of cases without AF is observed compared to the other group (54.7% versus 49.6%) (Chi2 = 38.819, *p* < 0.001). On the other hand, the proportion of patients with AF as a single diagnosis is significantly higher among those without dyslipidemia (29.2%) compared to those with dyslipidemia (23.1%). The proportion of AF accompanied by T2DM and/or CKD is higher among patients without dyslipidemia (11.3%) vs. others (7.8%). In the group of patients with dyslipidemia, on the other hand, cases of diabetes mellitus possibly associated with CKD are more frequent than in the other group (14.4% compared to 9.9%), Supplementary Materials (Figure S4, Tables S5 and S6). Regarding obesity, when we compared subjects with obesity (n = 1064) vs. normal weight (n = 447), we found that patients with higher BMI have a higher percentage of AF diagnosis (28.3% vs. 23.8%) and significantly higher rates are found in patients that have concomitant T2DM and/or CKD (13.2% vs. 8,6%) (Chi2 = 44.354, *p* < 0.001), Supplementary Materials (Table S7). These correspond with the current literature data that shows higher rates of obesity and metabolic syndrome in CKD and T2DM patients.

### 3.3. NLR Value, AF Association, and Comorbidities

By using the Mann–Whitney/Kruskal–Wallis test, the NLR value showed significative associations with male gender (U = 122,652.500, *p* < 0.001), with no significant correlation between NRL and environment (U = 130,654.500, *p* = 0.406) and age groups (H = 5.405, *p* = 0.067), Supplementary Materials (Table S8). NLR variates significantly in patients with AF and the comorbidities mentioned compared to subjects without the disease (H = 70.627, *p* < 0.001). Low values of NLR were found in patients without AF, with a mean value of 4.5188 ± 4.76883 and a median of 2.890. Significantly raised values of NLR were reported in subjects with AF diagnosis (4.7973 ± 4.06169) and a median of 3.6150, but also in AF+CKD, (5.5147 ± 4.08069, median 4.6540), and in the AF+CKD+T2DM group (6.9496 ± 4.31425). Moreover, the value of the NLR is also statistically significantly higher in patients with AF associated with CKD compared to those who only have AF but is lower, although insignificant, in patients who have AF and T2DM compared to those without this pathology (with a mean of 4.4451 ± 3.13192 and a median of 3.4444) (Table 2). Via Pairwise comparison between groups, NLR increases in patients with AF compared to those without pathology—absent vs. T2DM+AF (*p* = 1.000), absent vs. AF (*p* = 0.000), absent vs. CKD+AF (*p* = 0.000), absent vs. T2DM+CKD + AF (*p* = 0.002)—as well as in those with AF and CKD or AF and CKD + T2DM. This increase is significantly accentuated in the latter two groups (*p*-value of 0.003, *p* = 0.080, respectively). At the same time, the same ratio decreases slightly in patients with AF and T2DM compared to those without pathology, Supplementary Materials (Table S9).

### 3.4. PLR, AF, and Comorbidities

There is a significant statistical correlation between PLR and the presence of AF with comorbidities. However, in this case, some variations are worth mentioning. Firstly, AF patients have lower levels of average PLR (14.9284 ± 13.13541 with a median of 11.4054 vs. 15.6111 ± 14.65447 with a median of 11.0477) compared with non-AF patients; however, this is not statistically significant (Table 3). These results are similar in patients with AF and T2DM. On the other hand, in patients with AF associated with CKD, the PLR increases statistically significantly compared to the value recorded in patients without pathology, reaching an average of 18.5574 ± 16.89465, and a median of 13.3707 (*p* = 0.003). PLR value is higher in patients with AF, CKD, and T2DM (18.1729 ± 11.71315), but this time the difference from patients without pathology does not exceed the threshold of statistical significance (*p* = 1.000). Therefore, PLR is only significant statistically (*p* = 0.004) and raised in patients with AF+CKD, Supplementary Materials (Table S10).

### 3.5. Neutrophils, Lymphocytes, and Platelet Values in AF Patients

Lower neutrophil levels were found in patients without AF (66.860 ± 11.9606 and median 66.100). These values elevate significatively in subjects with AF (68.768 ± 10.9695, median of 69.100), AF+CKD (71.399 ± 10.5445, median of 72.200), and AF+CKD+T2DM (average of 74.591 ± 9.7488, median of 76.650) (*p* < 0.001). Patients with T2DM have higher levels of neutrophils than non-T2DM subjects; however, this time the difference is not statistically significant (absent vs. T2DM+AF, *p* = 0.807; AF vs. T2DM+AF, *p* = 1.000), Supplementary Materials (Tables S11 and S12).

The average value of lymphocytes in patients without AF (22.513 ± 10.1459, a median of 22.700) is significantly statistically lower than in patients with AF (H = 75.114, *p* < 0.001). Patients with only AF have a value of lymphocytes notable lower than those without pathology (20.383 ± 9.5604 and median 19.400, *p* = 0.000), a situation that is found similarly in the case of patients with AF and CKD, with a mean of 17.734 ± 9.0690 and median of 15.700, also in the case of AF, CKD, and T2DM (mean 14.691 ± 7.51692 and median 12.200). Although lymphocyte levels are more decreased in patients with AF and T2DM, the difference from patients without pathology is not statistically significant this time, because the medians in the two categories of patients are close, Supplementary Materials (Table S13 and S14).

On analysis, the mean value of platelets in patients without disease was 253.38 ± 89.741, with a median of 246.00. This value decreases statistically significantly in patients with AF without comorbidities (231.68 ± 87.618, median of 222.00) (H = 48.407, *p* < 0.001). The average platelet count is also low in other categories of patients with AF accompanied by diabetes or CKD. Still, in these cases, it is higher than in patients who only have AF, with an average value of around 242.00. The lowest values are found in patients with AF accompanied by diabetes mellitus and CKD, with an average of 206.95 ± 74.870; the median value is much lower than that observed in patients without pathology (204.50). However, the difference does not reach the statistical significance threshold (*p* = 1000), probably due to the small sample size of patients identified with all three pathologies (only 22 cases), Supplementary Materials (Tables S15 and S16).

### 3.6. ROC Analysis in AF Patients and Comorbidities

**Figure 2:**
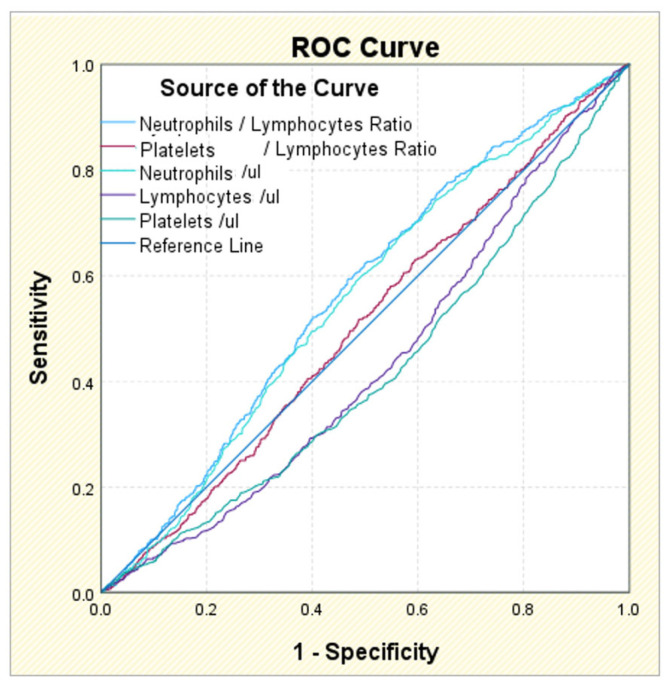
ROC curve of NLR, PLR, neutrophils, lymphocytes, and platelets in AF diagnoses.

**Figure 3:**
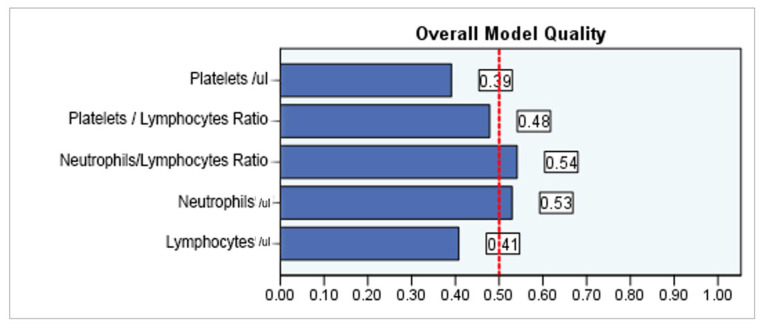
Quality of ROC models of NLR, PLR, neutrophils, lymphocytes, and platelets in AF diagnoses. The dashed line represents a non-discriminatory test, a random classifier, which assigns 1 and 0 randomly to the samples for model quality assessment.

The ROC analysis identified statistical significance for NLR (*p* = 0.000), but also for the separate values of neutrophils (*p* = 0.000), lymphocytes (*p* = 0.000), and platelets (*p* = 0.000) in AF diagnosis. However, the results are reserved. The area under the curve AUC analysis gave the best results for the NLR, with a coefficient of 0.565 for the cut-off value of 3.0570; the sensitivity associated with this value is 59.4%, but the specificity is 46.8%, below the minimum accepted threshold of 50%. The other marker for which the AUC coefficient is acceptable is represented by neutrophils, with a value of 0.554 associated with the cut-off value of 64.250; the sensitivity of this value is 67.0%, also acceptable, but the specificity is 43.9%, i.e., below the minimum required threshold of 50%. For lymphocytes and platelets, the AUC coefficients are below the threshold of 0.05, so the cut-off values obtained, although they have sensitivities of 98–99%, have almost zero specificities, and are therefore unusable (Table 4, Figure 2 and Figure 3).

Regarding the diagnosis of AF accompanied by diabetes, none of the five markers has a statistically significant association value. The area under the curve AUC is above the threshold of 0.5 for neutrophils, NLR, and PLR, but the significance coefficient is >0.05. The only marker with results at the limit of statistical significance is the neutrophil level, for which the ROC analysis cut-off value = 69,750; it has a sensitivity of 52.0%, which is weak but theoretically acceptable, and a specificity of 60.8%, which is also quite low, Supplementary Materials (Table S17).

In AF with CKD, all five biomarkers showed a statistically significant correlation. However, in this case, only NLR performed best, followed by the neutrophil level and PLR value. For NLR the AUC coefficient is 0.646, with a sensitivity of 60.7% and a specificity of 66.0% for the identified cut-off value of 3.9804. The next highlighted marker is the neutrophil level, with an AUC coefficient of 0.623 for the cut-off value of 67,150, which has a moderate sensitivity of 69.4%, although the specificity is reduced, at only 53.5%, but it is still above the minimum threshold of acceptability. The PLR, although it has an AUC coefficient of 0.578, i.e., still above the limit of 0.5, has a sensitivity below the acceptability threshold (49.5%) and a better specificity (64.4%) for the cut-off value of 13.7553, Supplementary Materials (Table S18).

In AF, diabetes mellitus, and CKD patients, both NLR and the neutrophil level had a moderate and predictive value. For the first marker, the AUC coefficient is 0.722, with a sensitivity of 63.6% and an even better specificity of 76.9%, for the cut-off value of 5.3302. The neutrophil level has an AUC coefficient of 0.689 for the cut-off value of 74.950, which is associated with a sensitivity of 63.6%, similar to the first marker, and a specificity of 74.0%, also acceptable. The other markers do not have satisfactory diagnostic potential, although it can be pointed out in the case of the PLR that the identified cut-off value, of 23.6151, has a specificity of 84.3%; however, the sensitivity is well below the acceptability threshold, being located at 36.4%, Supplementary Materials (Table S19).

## 4. Discussion

A gamut of research investigated the involvement of platelet, myeloid, and lymphoid precursors in acute and chronic non-inflammatory pathologies. Results showed that NLR and PLR individually or in combination may predict disease onset, help assess the risk of adverse events [16], and inform intervention follow-up and prognosis in AF [17] and non-cardiac diseases such as T2DM and CKD [18]. Existing studies have demonstrated that NLR and PLR serve as micro-inflammatory markers in patients with CKD; however, there is still debate about which is more sensitive in ascertaining inflammation [11].

Atrial stretch, ion channel dysfunction, cardiometabolic disturbances, inflammation, endothelial dysfunction, and autonomic dysregulation lead to atrial structure and electrophysiological changes that promote AF occurrence. Inflammation is a key driver of altered atrial electrophysiology and structure. Atrial cardiomyocyte hypertrophy, dedifferentiation, fibrosis, apoptosis, and myolysis are sustained by prolonged inflammation. Fibrosis is marked by overexpression of pro-inflammatory and pro-fibrotic cytokines that promote atrial remodeling (TGFβ1 and Galectin-3, TNF, interleukin-1β, and interleukin-6). Local inflammation is enhanced by visceral fat (pericardial adipose fat), also frequently found in T2DM and CKD patients. Interestingly, researchers have identified local responses that indicate an innate immune activity. These responses are expressed as higher CD45+ lymphocyte and CD68+ macrophage counts in the atrial wall, increased Toll-like receptors (TLRs), and MCP-1-induced protein activity [19].

Recent years have witnessed an increasing recognition of the complex role of neutrophil extracellular traps (NETs), mesh-like structures discharged by neutrophils, in CVD. NETs have a pivotal effect on shaping the immune response by activating other immune cells. They serve as a dynamic defense mechanism, are involved in tissue repair and remodeling, participate in autoimmune conditions, and act as dynamic players that actively participate in various aspects of the thrombotic process. Both experimental and clinical data suggest that AF patients have elevated NETs concentrations. In AF, NETs enhance myofibroblast differentiation, and fibrosis and promote a prothrombotic state [20]. An AF hypercoagulable state induces pro-fibrotic and pro-inflammatory responses in atrial fibroblasts. The location of the initial trigger of AF is generally not known, but pulmonary vein inflammation has been implicated [19]. More recently, the involvement of NLRP3 (NACHT, LRR, and PYD domain-containing protein 3) inflammasome in the atrium electric and structural remodeling has been described. AF electrical remodeling includes action potential shortening, reducing electrical connections between cells, and alterations in Ca2+ handling [21]. Mariani et al. [14] highlight the importance of identifying the most well-known determinants of AF to prevent disease onset and implement early, standardized, and cost-efficient treatment. Larger atrial size and decline in left ventricular function (HF) are a sign of atrial remodeling. The circadian rhythm is also a predictor of AF recurrence or spontaneous conversion. Nonetheless, the AF rate and duration, heart rate, blood pressure, and the number of past episodes of AF are major contributors to AF recurrence and persistence. Each patient evaluated should benefit from an accessible inflammatory panel that may identify and prevent AF appearance. Thus, early treatment may prevent thromboembolic adverse events and raise quality of life.

Previous evidence from the Framingham Heart Study [22] and the Norwegian Tromsø Study [23] indicated a positive correlation between complete blood count and AF. One systematic review and meta-analysis demonstrated the utility of hematological parameters (WBC, NLR, platelet count, and red blood cell distribution width) in predicting the occurrence and recurrence of AF [24]. Analysis of the Multi-Parameter Intelligent Monitoring in Intensive Care (MIMIC-IV) database concluded that NLR, PLR, and systemic immune-inflammatory index (SII) have the potential to serve as indicators for stratifying the risk of mortality in critically ill patients with AF [25]. In clinical practice, patients with heart conditions such as AF are often associated with additional comorbidities like T2DM [26] and CKD [27]. Therefore, we found it appropriate to investigate WBC markers in these patients. A few studies assessed NLR and PLR in patients with AF, T2DM, and/or CKD [28]; however, we could not find a study that included analysis across all three diseases. 

Our results showed that AF is more prevalent in males than in women, and its incidence rises with age. Also, subjects over 60 years old are more prone to develop and be diagnosed, with CKD and/or T2DM. These results are consistent with previous reports from The Global Burden of Disease (GBD). These disparities may be attributed to sex-specific variations in AF risk factors [29]. There is a paucity of research on sex-specific differences; males and females differ in sex hormone characteristics, immune reaction, and CVD expression, which influence NLR values and their variations along the life course [30]. Most AF, CKD, T2DM, and the combination of these diseases had higher rates in rural patients. This pattern can be attributed to the fact that the countryside population is less informed, underdiagnosed, and has less access to healthcare services.

Among the most frequent comorbidities, dyslipidemia and obesity are often observed in patients with cardiac arrhythmia, T2DM, and CKD. We have found that in our group of individuals, a statistically significantly higher proportion of cases without AF is linked to dyslipidemia compared to patients with AF (Chi2 = 38.819, *p* < 0.001). Furthermore, dyslipidemia as a comorbidity was identified in a higher proportion of T2DM and CKD subjects. Diabetes and CKD, through common and intricate pathways (i.e., hyperglycemia, insulin resistance, endothelial dysfunction, oxidative stress, lipid metabolism abnormalities, increased free fatty acids, albuminuria/proteinuria), are more susceptible to developing dyslipidemia. Dyslipidemia, in turn, is associated with the occurrence and progression of diabetic nephropathy and other atherosclerotic CVD complications. Additionally, obesity is a prevalent risk factor for metabolic diseases, characterized by a pro-inflammatory state and elevated oxidative stress. These changes lead to endothelial dysfunction and a hypercoagulable state [31]. Accordingly, we found that patients with a higher BMI have a greater percentage of AF, with significantly higher rates in patients with concomitant T2DM and/or CKD (*p* < 0.001). 

NLR was among the first biomarkers that were easily obtained and examined for predictive and prognostic roles; high values (>5) were associated with cardiovascular death, all-cause mortality, and net clinical outcomes [32]. Kundnani et al. demonstrated NLR’s utility as a biomarker to predict in-hospital mortality in patients with acute decompensated AF, showing the ability to predict the risk of death with a sensitivity of 72.8% and a specificity of 70.4% [33]. In this analysis, NLR was associated with male gender (*p* < 0.001), while no significant correlation was found with the environment (*p* = 0.406), as well as age groups (*p* = 0.067). Previous research established that markers of inflammation such as NLR and PLR are influenced by demographic and behavioral characteristics, suggesting that different cutoff points should be set according to social factors [34].

Relating to AF, NLR associated statistical significance (*p* = 0.000), with a cut-off value of 3.0570, and high sensitivity but low specificity. Significantly raised values of NLR were reported in subjects with AF than in those without cardiac arrhythmia in all groups. Interestingly, slightly higher values were determined in AF vs. AF+T2DM and in AF+CKD and AF+T2DM, although insignificant statistically. In contrast, others showed that NLR levels were associated with an increased risk of developing T2DM even after adjusting for multiple confounding factors (*p* = 0.003). NLR values differed between T2DM and non-T2DM patients (4.189 ± 4.154 vs. 4.095 ± 8.851, *p* = 0.009) [28]. Other studies also noted that high NLR is correlated positively with CKD stage [35,36] and may predict renal disease progression (median NLR was 2.76) [37]. In a cross-sectional study, Lin et al. [38] demonstrated NLR association with a higher CKD risk in overweight/obese men (*p* = 0.004) and women (*p* = 0.0090), and no correlation in normal-weight subjects.

PLR, another studied biomarker, has gained attention in CVD, as it integrates two opposite pathways: thrombotic and inflammatory. Enhanced platelet activation is encountered in diabetes. It also plays a pivotal role in the pathogenesis of glomerular disease via inflammation, coagulation, and fibrosis [39]. Mostly evaluated in patients with AF post-interventions such as coronary artery bypass graft surgery (CABG), researchers found a significant association between AF and PLR [40,41,42]. Fan et al. [43] showed that PLR combined with the CHA_2_DS_2_-VAS_c_ score are independent risk factors for cardiogenic cerebral embolism (CCE) in patients with nonvalvular AF. After a mean follow-up of 15.1 ± 9.3 months, PLR was an independent predictive factor of long-term AF recurrence post ablation, especially in nonparoxysmal AF, and improved APPLE score predictability [44]. In addition, recently elevated PLR has been independently associated with atrial tachyarrhythmia in patients with other comorbidities [45]. Thus, PLR has a predictive and prognostic role in AF and in AF-related disease. Our results found that PLR is independently and significantly associated with AF diagnosis (*p* = 0.004, cut off = 9.5982) and in patients with AF+CKD. Patients with AF, CKD, and T2DM had higher levels of PLR than other groups; however, due to the small sample size, the results had no significant statistical value. However, a larger sample size of AF and comorbidities is needed to demonstrate PLR’s specific role.

When analyzing the parameters individually, lowered levels of neutrophils were identified in non-AF subjects, while higher levels were found in each group of patients with diseases. A cut-off value of 64.250 for AF diagnosis was identified. Neutrophils and neutrophil extracellular traps (NETs) more specifically, induce autophagic apoptosis of cardiomyocytes through the induction of mitochondrial injury, via ADP decrease by promoting the progression of AF and AF-related fibrosis [46,47]. The current literature exhibits similar results; these powerful mediators of inflammation have highly adaptable and versatile functions and are raised in CKD and T2DM [48]. On the other hand, lymphopenia often indicates physiological stress, suppression of the immune system, and worsening health. Lymphocytes play different roles in AF and the combination of different lymphocytes in AF constitutes the protective effect of their total count in AF [49]. We found that patients with only AF, or those with AF and T2DM or CKD, have significantly lower levels of lymphocytes compared with patients without the disease (*p* < 0.001). Yen et al. [50] demonstrated that in individuals with CKD (n = 2128), differential leukocyte counts, including neutrophil and lymphocyte, were higher in CKD patients than the non-CKD population (*p* < 0.001). These results confirmed that hyperinflammation was prominent in renal disease. Finally, platelet counts are linked to CVD and non-CVD. Their values are linked with a higher risk of thrombotic diseases, the formation of atherosclerotic plaques, and increased malignant cardiovascular events [51]. Our results showed that patients with AF have lower levels of platelets than non-AF patients. These results are reflected in all groups: AF, T2DM, and/or CKD.

Although in all individual neutrophils, lymphocytes, platelets, and NLR are significantly associated with AF, the stability of NLR is better and less affected by physiological and pathological status. The ROC value of NLR was better compared to PLR in diagnosing diseases. Regarding the diagnosis of AF accompanied by diabetes, none of the five markers have a statistically significant association value. Li et al. [52] showed that NLR compared to other inflammatory markers (PLR or monocyte to lymphocyte ratio) has more potential in identifying the risk of T2DM and diabetic kidney disease. In AF with CKD, all five biomarkers showed a statistically significant correlation. However, in this case, only NLR performed best, followed by the neutrophils level and PLR value. In AF, T2DM, and CKD patients, both NLR (cut-off value of 5.3302) and the neutrophil (cut-off value of 74.950) level had a moderate and predictive value.

The results from studies over the last two decades concluded that a normal range of NLR is between 1 and 3, while PLR varies between 90 and 210. An NLR between 2.3 and 3.0 is a grey zone and may serve as an early warning of a pathological state, while a value lower than 0.7 or higher than 3.0 is considered pathological. Currently, NLR and PLR can be easily calculated online. The NLR has a good discriminant function for evaluating patients for possible acute infections, as well as for prognostication of several conditions, including pulmonary embolism. However, a general cut-off is difficult to assess due to their heterogeneity. Additionally, every inflammatory response is somewhat different, depending on the underlying disease and its status, whether acute or chronic.

The values of NLR and PLR vary depending on the disease status of the patients and the timing of determination. For example, when comparing the cut-off values of NLR in AF (3.06) established in this study with those in the literature, our values are higher than others. Shao et al. [53] meta-analysis found cut-off values of NLR in incident AF of 1.25 and in post-AF of 1.518. These differences can be attributed to the fact that permanent and persistent AF demonstrate a higher pro-inflammatory state than newly diagnosed AF. Additionally, we found values of 3.9804 in AF with CKD and 5.3302 in AF +T2DM, indicating that a higher inflammatory status may characterize AF and diabetes. We only identified the study by Cheng et al. [54], which evaluated NLR values in patients with AF and T2DM. They showed that NLR increases at follow-up assessment and is associated with the risk of stroke in these patients, though no precise cut-off values are mentioned. Regarding CKD, we found studies that demonstrated the predictive power of NLR and PLR in diabetic kidney disease diagnosis [55], but none that investigated both markers in AF and CKD for comparison. Further studies are needed in AF and comorbidities to enable a comprehensive comparison. Nevertheless, these markers have shown their predictive value in disease diagnosis, indicating a pro-inflammatory state. The greater the change in value, the higher the assumed risk of CVD adverse events and poor prognosis. Moreover, these results are attributed to differences in sample size between groups, as well as other factors such as disease severity and medication administration. Thus, our study has some limitations. 

Firstly, being a retrospective study, we could not acquire the stages of each disease, and we could not grade obesity as our data was retrieved from the ICD-10 codes. This can influence the complete blood count values. Secondly, as the sample size is from a single center, regarding socioeconomic factors, race, and culture, our study participants may not be representative of the general population, and taking account of the geographical heterogeneity, external validation should be performed to exclude selection bias.

## 5. Conclusions

The role of white and red blood cell counts in assisting clinical decision-making in CVD and non-CVD is still unclear. While these tests are reliable, accessible, and cost-effective, their standalone predictive performance for a specific disease is limited. Nonetheless, accumulating evidence suggests that NLR, PLR, and especially their combination can provide valuable information to clinicians who encounter multi-comorbidity patients. This study shows that NLR and PLR are associated with AF, CKD, and T2DM with different cut-off values, and highlights the shifts in platelet, lymphocyte, and neutrophil levels in these conditions. These results and our conclusions need to be validated in larger prospective studies.

## Figures and Tables

**Table 1:** WBC count, NLR, PLR, and other laboratory parameters in AF patients.

	N	Mean	Std. Deviation	Min	Max	Median	IQR
							25th–75th
Days of hospital	1084	6.25	3.00	0.00	30.00	6.00	4.00–7.00
NLR	1066	4.95	4.03	0.19	38.25	3.74	2.44–5.95
PLR	1066	15.66	13.79	1.74	172.97	11.64	7.94–18.12
Neutrophils ×10^3^/µL	1066	69.40	10.92	15.30	93.00	70.10	62.50–77.10
Lymphocytes ×10^3^/µL	1066	19.79	9.44	2.40	80.0	18.70	12.90–25.83
Platelet ×10^3^/µL	1067	234.10	88.65	32.00	780.00	223.00	180.00–274.00
Hb (g/dL)	960	12.90	2.40	3.40	19.60	13.20	11.80–14.40
Leucocytes ×10^3^/µL	1066	12.45	55.92	0.00	1406.60	831	6.42–10.42
CRP (mg/dL)	938	2.62	4.71	0.02	42.84	0.92	0.36–2.66
Fibrinogen (mg/dL)	644	361.87	91.23	140.00	816.00	356.50	303.00–415.00
Na (mmol/L)	1078	138.43	5.10	104.00	149.00	140.00	137.00–141.00
K (mmol/L)	1016	4.45	0.61	2.30	7.40	4.40	4.10–4.80
Urea (mg/dL)	1080	48.83	25.50	11.00	189.00	42.00	33.00–57.00
Creatinine (mg/dL)	1079	1.02	0.42	0.38	5.49	0.91	0.76–1.14
HbA1c (%)	558	6.66	1.49	3.90	14.50	6.20	5.70–7.20
CK (mg/dL)	772	150.27	508.46	15.00	11,087.00	78.00	51.00–133.75
CK-MB (mg/dL)	655	25.64	31.44	6.00	431.00	20.00	15.00–27.00
NT-proBNP (pg/mL)	170	5081.77	5879.78	93.10	28,839.00	2718.00	1191.75–6527.25
hsTnI (ng/mL)	136	40.99	117.55	0.00	1009.00	10.05	6.29–25.55
Total cholesterol (mg/dL)	996	152.48	46.81	40.00	382.00	148.00	119.00–180.75
LDL (mg/dL)	309	94.19	37.73	17.00	209.00	89.00	66.00–118.00
HDL (mg/dL)	985	40.14	15.80	5.00	146.00	38.00	29.00–49.00
TG (mg/dL)	987	106.79	57.97	28.00	679.00	93.00	71.00–126.00
Ferritin (mg/dL)	713	253.64	1387.84	5.00	35,441.00	111.00	59.00–216.00
Iron (mg/dL)	770	65.37	37.39	6.00	253.00	57.50	38.00–83.25
AST (U/L)	983	44.76	116.53	7.00	1573.00	24.00	19.00–33.00
ALT (U/L)	1061	39.34	88.53	4.00	1829.00	22.00	16.00–34.00

NLR—neutrophil-to-lymphocyte ratio; PLR—platelet-to-lymphocyte ratio; CRP—C-reactive protein; Hb—hemoglobin; Na—sodium; K—potassium; CK—creatine kinase; CK-MB—creatine phosphokinase–MB; hsTnI—high-sensitivity troponin I; TG—triglyceride; ALT—alanine transaminase; AST—aspartate transaminase; mean ± SD.

**Table 2:** Comparative values of the NLR depend on the presence of AF, with/without comorbidities via the Kruskal–Wallis H test.

NLR							IQR		Kruskal–Wallis H Test
	N	Mean	Std. Deviation	Min	Max	Median	25th	75th	
Score AF + Comorbidities									
Absent	1478	4.52	4.77	0.34	68.50	2.89	2.00	5.05	H = 70.627
AF	773	4.80	4.06	0.21	38.25	3.62	2.38	5.53	*p* < 0.001 **
T2DM+AF	75	4.45	3.13	0.95	15.64	3.44	2.32	5.95	
CKD+AF	196	5.51	4.08	0.19	30.07	4.56	2.83	6.71	
T2DM+CKD+AF	22	6.95	4.31	1.99	17.06	6.43	3.04	9.04	

T2DM-type 2 diabetes mellitus, CKD-chronic kidney disease, AF-atrial fibrillation, *p* < 0.003 **—high significative statistic.

**Table 3:** Comparative values of the PLR depend on the presence of AF, with/without comorbidities via the Kruskal–Wallis H test.

NLR							IQR		Kruskal–Wallis H Test
	N	Mean	Std. Deviation	Min	Max	Median	25th	75th	
AF + Comorbidities									
Absent	1478	15.61	14.65	0.24	173.68	11.05	7.68	17.51	H = 15.478
AF	773	14.92	13.13	1.74	172.97	11.41	7.74	16.70	*p* = 0.004 **
T2DM+AF	75	14.89	10.76	3.04	64.14	11.02	7.89	19.27	
CKD+AF	196	18.55	16.89	3.14	135.52	13.37	8.90	22.00	
T2DM+CKD+AF	22	18.17	11.72	5.98	46.11	13.46	8.66	26.25	

T2DM—type 2 diabetes mellitus; CKD—chronic kidney disease; AF—atrial fibrillation; *p* < 0.001 **—high significative statistic.

**Table 4:** ROC analysis of NLR, PLR, neutrophils, lymphocytes, and platelets in AF diagnoses.

Diagnostic: AF	AUC	*p*-Value	95% CI		Gini Index	Cut-Off Value	Sensitivity	Specificity
			L.inf	L.sup				
Neutrophil/Lymphocyte Ratio	0.565	0.000 **	0.541	0.590	0.131	**3.06**	0.594	0.468
Platelet/Lymphocyte Ratio	0.503	0.801	0.478	0.528	0.006	**9.60**	0.630	0.406
Neutrophils/μL	0.554	0.000 **	0.530	0.579	0.108	**64.25**	0.670	0.439
Lymphocytes/μL	0.432	0.000 **	0.408	0.457	−0.136	**4.95**	0.986	0.029
Platelets/μL	0.416	0.000 **	0.391	0.440	−0.169	**73.00**	0.992	0.011

AF—atrial fibrillation; *p* < 0.001 **—high significative statistic.

## Data Availability

The original contributions presented in this study are included in the article. Further inquiries can be directed to the corresponding author.

## Supplementary Materials

The following supporting information can be downloaded at: https://www.mdpi.com/article/10.3390/life15060960/s1.
