# Supplementary material for: The Role of Neutrophil-to-Lymphocyte Ratio and Platelet-to-Lymphocyte Ratio in Predicting Atrial Fibrillation and Its Comorbidities
Source: Life (Basel). 2025 Jun 16;15(6):960. doi: 10.3390/life15060960 (PMC12193945; doi:10.3390/life15060960)
Supplement: Supplementary file 1 [file life-15-00960-s001.zip › life-3651308-supplementary.pdf]

SUPPLEMENTARY S1.

Table S1. Distribution of AF patients according to demographic characteristics, sex, age groups, and environment.

		Frequency	Percent	Valid Percent	Cumulative Percent
		**Sex**			
**Valid**	male	576	53.1	53.1	
	female	508	46.9	46.9	
	Total	1084	100.0	100.0	
		**Age**			
**Valid**	< 40 years	7	0.6	0.6	
	40-60 years	162	14.9	14.9	
	> 60 years	915	84.4	84.4	
	Total	1084	100.0	100.0	
		**Environment**			
**Valid**	U-419	38.7	38.7	38.7	
	R- 665	61.3	61.3	100.0	
	T-1084	100.0	100.0		

[1]*U—urban, R-rural, T-Total;*

Figure S1. Distribution of patients according to the presence of Atrial Fibrillation accompanied by comorbidities – comparative study by sex.

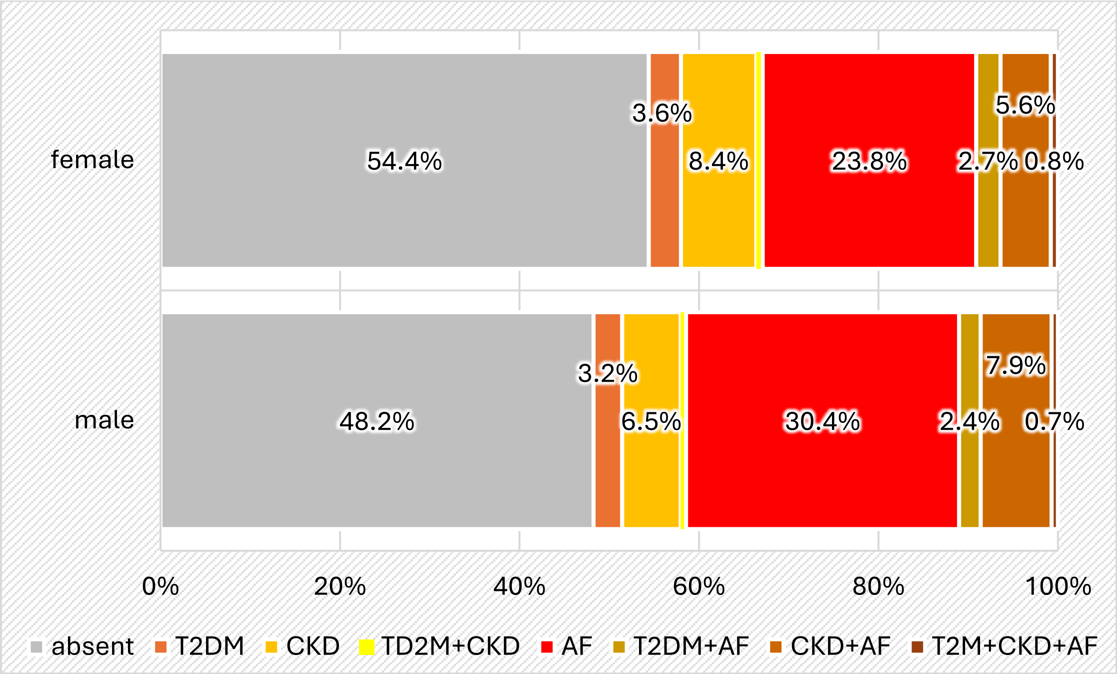

[1]*T2DM-type 2 diabetes mellitus, CKD-chronic kidney disease, AF-atrial fibrillation ;*

Table S2. Distribution of patients according to the presence of Atrial Fibrillation accompanied by comorbidities – comparative study by sex.

**AF and Comorbidities**	**Sex**				Total		Pearson Chi-squared test
	**male**		**female**				
	N	%	N	%	N	%	
absent	670	48.2%	841	54.4%	1511	51.5%	Chi2 = 27.092
T2DM	45	3.2%	55	3.6%	100	3.4%	*p <0.001***
CKD	90	6.5%	130	8.4%	220	7.5%	
T2DM + CKD	8	0.6%	11	0.7%	19	0.6%	
AF	422	30.4%	368	23.8%	790	26.9%	
AF + T2DM	34	2.4%	41	2.7%	75	2.6%	
AF + CKD	110	7.9%	87	5.6%	197	6.7%	
AF + TDM + CKD	10	0.7%	12	0.8%	22	0.7%	
**Total**	**1389**	**100.0%**	**1545**	**100.0%**	**2934**	**100.0%**	

[2] *T2DM-type 2 diabetes mellitus, CKD-chronic kidney disease, AF-atrial fibrillation, p<0.001**.*

Figure S2. Distribution of patients according to the presence of Atrial Fibrillation accompanied by comorbidities – comparative study by age groups.

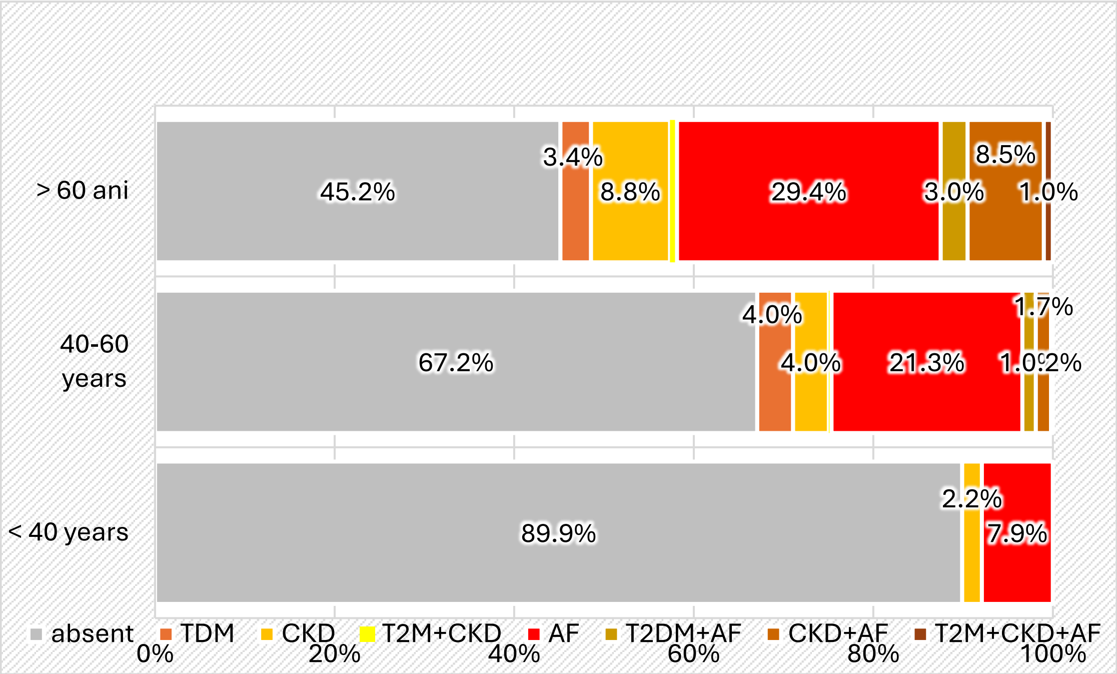

[2] *T2DM-type 2 diabetes mellitus, CKD-chronic kidney disease, AF-atrial fibrillation.*

Table S3. Distribution of patients according to the presence of Atrial Fibrillation accompanied by comorbidities – comparative study by age groups.

	Age groups						Total		Pearson Chi-squared test
	< 40 years		40-60 years		> 60 years				
**AF and Comorbidities**	N	%	N	%	N	%	N	%	
absent	80	89.9%	442	67.2%	989	45.2%	1511	51.5%	Chi2 = 176.376
T2DM			26	4.0%	74	3.4%	100	3.4%	*p <0.001***
CKD	2	2.2%	26	4.0%	192	8.8%	220	7.5%	
T2DM + CKD			2	0.3%	17	0.8%	19	0.6%	
AF	7	7.9%	140	21.3%	643	29.4%	790	26.9%	
T2DM + AF			10	1.5%	65	3.0%	75	2.6%	
CKD + AF			11	1.7%	186	8.5%	197	6.7%	
T2DM + CKD + AF			1	0.2%	21	1.0%	22	0.7%	
**Total**	**89**	**100.0%**	**658**	**100.0%**	**2187**	**100.0%**	**2934**	**100.0%**	

[3] *T2DM-type 2 diabetes mellitus, CKD-chronic kidney disease, AF-atrial fibrillation, p<0.001**.*

Figure S3. Distribution of patients according to the presence of Atrial Fibrillation accompanied by comorbidities – comparative study environment.

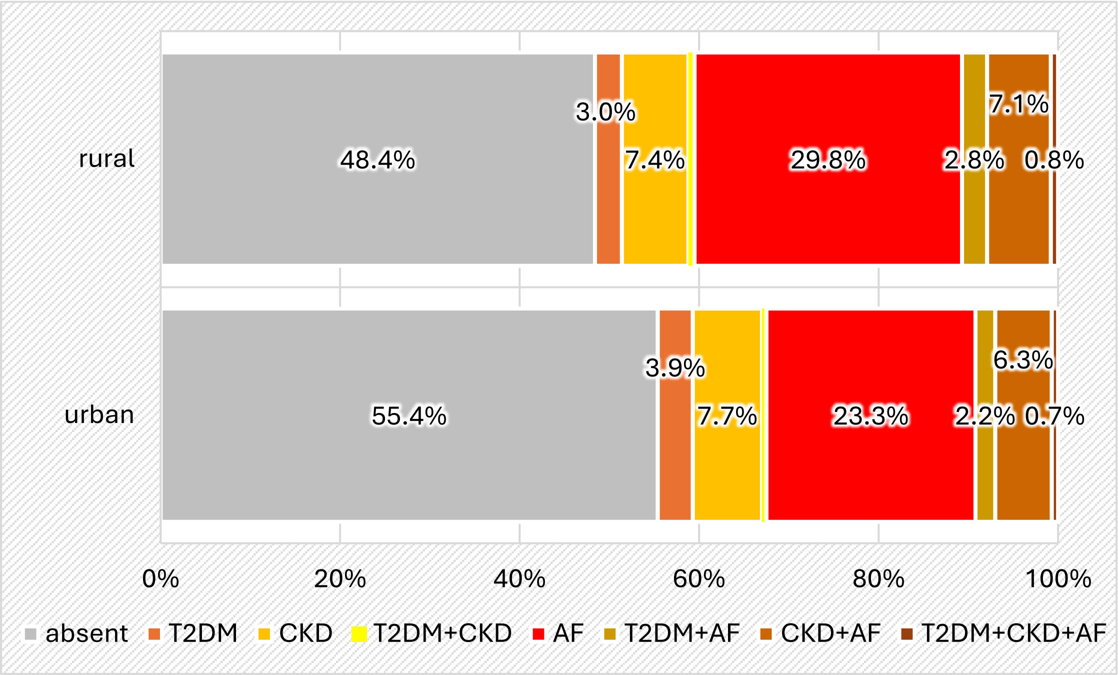

[3] *T2DM-type 2 diabetes mellitus, CKD-chronic kidney disease, AF-atrial fibrillation.*

Table S4. Distribution of patients according to the environment and the presence of Atrial Fibrillation and comorbidities – comparative study.

**AF+comorbidities**	**Mediu**				Total		Pearson Chi-squared test
	**urban**		**rural**				
	N	%	N	%	N	%	
absent	715	55.4%	796	48.4%	1511	51.5%	Chi2 = 21.961
T2DM	50	3.9%	50	3.0%	100	3.4%	*p = 0.003***
CKD	99	7.7%	121	7.4%	220	7.5%	
T2DM + CKD	7	0.5%	12	0.7%	19	0.6%	
AF	300	23.3%	490	29.8%	790	26.9%	
T2DM+ AF	29	2.2%	46	2.8%	75	2.6%	
CKD + AF	81	6.3%	116	7.1%	197	6.7%	
T2DM+CKD+AF	9	0.7%	13	0.8%	22	0.7%	
**Total**	**1290**	**100.0%**	**1644**	**100.0%**	**2934**	**100.0%**	

[4] *T2DM-type 2 diabetes mellitus, CKD-chronic kidney disease, AF-atrial fibrillation, p<0.003**-high significative statistic.*

Table S5. Cumulative percentage of patients according to dyslipidemia and obesity.

		**Frequency**	**Percent**	**Valid Percent**	**Cumulative Percent**
			**Dyslipidemia**		
**Valid**		743	68.5	68.5	68.5
		341	31.5	31.5	100.0
		1084	100.0	100.0	
			**Obesity**		
**Valid**		747	68.9	68.9	68.9
		337	31.1	31.1	100.0
		1084	100.0	100.0	

Table S6. *Distribution of patients according to the presence of Atrial Fibrillation comorbidities and dyslipidemia a comparative analysis.*

**AF+comorbidities**	**absent**		**present**		**Total**		**Pearson Chi-squared test**
	N	%	N	%	N	%	
absent	907	49.6%	604	54.7%	1511	51.5%	Chi2 = 38.819
T2DM	47	2.6%	53	4.8%	100	3.4%	p <0.001**

	CKD	122	6.7%	98	8.9%	220	7.5%
	T2DM + CKD	11	0.6%	8	0.7%	19	0.6%
	AF	535	29.2%	255	23.1%	790	26.9%
	T2DM+ AF	48	2.6%	27	2.4%	75	2.6%
	CKD + AF	143	7.8%	54	4.9%	197	6.7%
	T2DM+CKD+AF	17	0.9%	5	0.5%	22	0.7%
**Total**		**1830**	**100.0%**	**1104**	**100.0%**	**2934**	**100.0%**

*[6]T2DM-type 2 diabetes mellitus, CKD-chronic kidney disease, AF-atrial fibrillation, p<0.001** - high significative statistic;*

Figure S4. Distribution of patients according to the presence of Atrial Fibrillation comorbidities and dyslipidemia a comparative analysis.

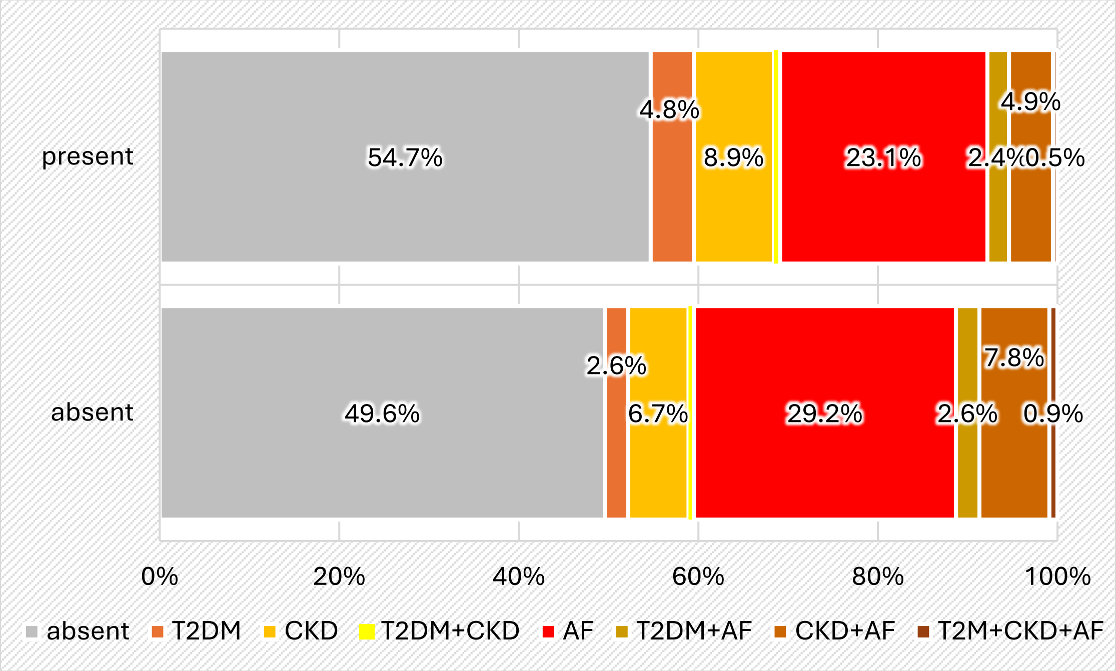

[4] T2DM-type 2 diabetes mellitus, CKD-chronic kidney disease, AF-atrial fibrillation.

Table S7. Distribution of patients according to the presence of Atrial Fibrillation comorbidities and obesity a comparative analysis.

**AF + comorbidities**	**OBESITY**	Total

	absent		present				Pearson Chi-squared test
	N	%	N	%	N	%	
absent	1064	52.6%	447	49.1%	1511	51.5%	Chi2 = 44.354
T2DM	55	2.7%	45	4.9%	100	3.4%	*p <0.001***
CKD	145	7.2%	75	8.2%	220	7.5%	
T2DM + CKD	13	0.6%	6	0.7%	19	0.6%	
AF	573	28.3%	217	23.8%	790	26.9%	
T2DM+ AF	33	1.6%	42	4.6%	75	2.6%	
CKD + AF	131	6.5%	66	7.3%	197	6.7%	
T2DM+CKD+AF	10	0.5%	12	1.3%	22	0.7%	
**Total**	**2024**	**100.0%**	**910**	**100.0%**	**2934**	**100.0%**	

*[7] T2DM-type 2 diabetes mellitus, CKD-chronic kidney disease, AF-atrial fibrillation, p<0.001** - high significative statistic.*

Table S8. NLR association depends on sex, environment, and age groups.

NLR (n=1066)	N	Mean	Std. deviation	Min	Max	Median	IQR 25th	IQR 75th	Mann-Whitney / Kruskal-Wallis test
**Sex**									
male	569	5.28	4.21	0.46	38.25	4.04	2.51	6.40	U = 122652.500
female	497	4.57	3.78	0.19	30.07	3.43	2.32	5.44	*p<0.001***
**Environment**									
urban	412	5.17	4.43	0.19	38.25	3.78	2.44	6.17	U = 130654.500
rural	654	4.81	3.75	0.21	30.07	3.73	2.45	5.84	*p = 0.406*
**Age groups**									
< 40 years	7	5.54	3.48	2.38	12.63	5.16	2.67	6.40	H = 5.405
40-60 years	159	4.34	3.48	0.95	28.63	3.40	2.24	5.03	*p = 0.067*
> 60 years	900	5.05	4.12	0.19	38.25	3.78	2.45	6.03	

*[8] p<0.001**- high significative statistic.*

Table S9. Pairwise comparison analysis for NLR in non-AF and AF patients with/without comorbidities.

**Pairwise Comparisons of AF + comorbidities**	Test Statistic	*p-value*
Absent vs. T2DM+AF	-134.65	1.000
Absent vs. AF	-164.88	0.000**
Absent vs. CKD+ AF	-377.86	0.000**
Absent vs. T2DM + CKD + AF	-586.20	0.002**
T2DM + AF vs. AF	30.23	1.000
T2DM + AF vs. CKD + AF	-243.22	0.147

T2DM + AF vs. T2DM + CKD + AF	-451.56	0.112
AF vs. CKD + AF	-212.99	0.003**
AF vs. T2DM +CKD+ AF	-421.33	0.080
CKD + AF vs. T2DM + CKD + AF	-208.34	1.000

[9] *T2DM-type 2 diabetes mellitus, CKD-chronic kidney disease, AF-atrial fibrillation, p<0.001** - high significative statistic.*

Table S10. Pairwise comparison study for PLR-AF with/without comorbidities

**Pairwise Comparisons of AF + comorbidities**	Test Statistic	*p-value*
Absent vs. AF	-7.39	1.000
Absent vs. T2DM+AF	-48.40	1.000
Absent vs. CKD+AF	-201.45	0.003**
Absent vs. T2DM+CKD+AF	-246.41	1.000
AF vs. T2DM+AF	-41.02	1.000
AF vs. CKD+AF	-194.06	0.010*
AF vs. T2DM+CKD+AF	-239.03	1.000
T2DM+AF vs. CKD+AF	-153.05	1.000
T2DM+AF vs. T2DM+CKD+AF	-198.01	1.000
CKD+AF vs. T2DM+CKD+AF	-44.97	1.000

[10] *T2DM-type 2 diabetes mellitus, CKD-chronic kidney disease, AF-atrial fibrillation, p<0.001** - high significative statistic.*

Table S11. Neutrophil levels in patients with/ without AF and comorbidities

**Neutrophils**	N	Mean	Std. deviation.	Min	Max	Median a	IQR 25th	IQR 75th	Kruskal-Wallis H test
**Score AF+ comorbid**									
absent	1478	66.86	11.96	9.50	95.90	66.10	58.58	75.30	H = 50.444
AF	773	68.77	10.97	16.90	92.90	69.10	62.05	76.40	*p <0.001***
T2DM+AF	75	69.14	10.80	42.40	93.00	70.30	62.30	78.20	
CKD+AF	196	71.40	10.55	15.30	92.30	72.20	64.60	78.60	
T2DM+CKD+AF	22	74.59	9.75	56.80	87.00	76.65	64.43	83.23	

[11] *T2DM-type 2 diabetes mellitus, CKD-chronic kidney disease, AF-atrial fibrillation, p<0.001** - high significative statistic.*

Table S12. Neutrophil levels in patients with/ without AF and comorbidities- Pairwise Comparisons

Pairwise Comparisons of Scor Aritmii + comorbid	Test Statistic	*p-value*
Absent vs. AF	-136.09	0.000**
Absent vs. T2DM+AF	-151.85	0.807
Absent vs. CKD+AF	-317.51	0.000**
Absent vs. T2DM+CKD+AF	-503.97	0.014*
AF vs. T2DM+AF	-15.76	1.000
AF vs. CKD+AF	-181.42	0.020*
AF vs. T2DM+CKD+AF	-367.88	0.205
T2DM+AF vs. CKD+AF	-165.66	0.967
T2DM+AF vs. T2DM+CKD+AF	-352.12	0.480
CKD+AF vs. T2DM+CKD+AF	-186.46	1.000

[25] *T2DM-type 2 diabetes mellitus, CKD-chronic kidney disease, AF-atrial fibrillation, p<0.001** - high significative statistic.*

Table S13. Lymphocyte levels in patients with/ without AF and comorbidities

Lymfocyte	N	Mean	Std. deviation	Min	Max	Median a	IQR 25th	IQR 75th	Kruskal-Wallis H test
**AF + comorbidities**									
absent	1478	22.52	10.15	1.40	69.40	22.70	14.80	29.20	H = 75.114
AF	773	20.38	9.56	2.40	80.00	19.40	13.55	26.20	*p <0.001***
TDM+AF	75	20.58	8.38	5.80	45.00	20.40	12.50	26.50	
CKD+AF	196	17.73	9.07	2.90	79.30	15.70	11.70	22.65	
T2DM+CKD+AF	22	14.69	7.52	5.10	28.60	12.20	9.23	21.43	

[13] *T2DM-type 2 diabetes mellitus, CKD-chronic kidney disease, AF-atrial fibrillation, p<0.001** - high significative statistic.*

Table S14. Lymphocyte levels in patients with/ without AF and comorbidities-Pairwise Comparisons.

Pairwise Comparisons of Scor AF + comorbidities	Test Statistic	*p-value*
T2DM+CKD+ AF vs. CKD +AF	215.11	1.000
T2DM +CKD + AF vs. AF	433.60	0.063
T2DM + CKD +AF vs. T2DM + AF	473.41	0.079
T2DM + CKD + AF vs. absent	604.42	0.001**
CKD +AF vs. AF	218.49	0.002**
CKD + AF vs. T2DM + AF	258.30	0.096
CKD + AF vs. absent	389.31	0.000**
AF vs. T2DM + AF	-39.82	1.000

AF vs. absent	170.83	0.000**
T2DM + AF vs. absent	131.01	1.000

[14] *T2DM-type 2 diabetes mellitus, CKD-chronic kidney disease, AF-atrial fibrillation, p<0.001** - high significative statistic.*

Table S15. Platelet levels in patients with/ without AF and comorbidities

Platelets	N	Mean	Std. Deviation	Min	Max	Median	IQR		Kruskal-Wallis H test
							25th	75th	
**AF+ comorbidities**									
absent	1478	253.38	89.74	11.00	821.00	246.00	198.00	296.00	H = 48.407
AF	773	231.68	87.62	32.00	780.00	222.00	176.50	273.00	p <0.001**
T2DM+AF	75	243.64	85.36	82.00	600.00	223.00	191.00	274.00	
CKD+AF	196	242.97	94.47	62.00	702.00	227.00	184.00	294.00	
T2DM+CKD+AF	22	206.95	74.87	94.00	365.00	204.50	139.25	264.50	

[15] *T2DM-type 2 diabetes mellitus, CKD-chronic kidney disease, AF-atrial fibrillation, p<0.001** - high significative statistic.*

Table S16. Platelet levels in patients with/ without AF and comorbidities-Pairwise Comparisons.

**Pairwise Comparisons of Scor Aritmii + comorbid**	Test Statistic	*p-value*
T2DM +CKD + AF vs. AF	181.88	1.000
T2DM+CKD+ AF vs. CKD +AF	269.12	1.000
T2DM + CKD +AF vs. T2DM + AF	275.68	1.000
T2DM + CKD + AF vs. absent	396.13	0.121
AF vs. CKD+AF	-87.24	1.000
AF vs. T2DM+AF	-93.79	1.000
AF vs. absent	214.24	0.000**
CKD+AF vs. T2DM+AF	6.56	1.000
CKD+AF vs. absent	127.01	0.227
T2DM+AF vs. absent	120.45	1.000

[16] *T2DM-type 2 diabetes mellitus, CKD-chronic kidney disease, AF-atrial fibrillation, p<0.001** - high significative statistic.*

Table S17. ROC analysis of NLR, PLR, neutrophils, lymphocytes and platelets in AF+T2DM diagnosis

**Diagnostic:** **T2DM + AF**	AUC	*p-value*	95% CI L.inf	95% CI L.sup	Gini Index	Cut-off value	Sensitivity	Specificity
Neutrophils / Lymphocyte Ratio	0.555	0.081	0.493	0.616	0.109	**3.13**	0.587	0.541
Platelet / Lymphocyte Ratio	0.519	0.571	0.454	0.584	0.038	**14.82**	0.413	0.677
Neutrophils /ul	0.560	0.057	0.498	0.622	0.121	**69.75**	0.520	0.608
Lymphocytes /ul	0.446	0.080	0.385	0.506	-0.108	**9.15**	0.947	0.106
Platelet /ul	0.450	0.131	0.386	0.515	-0.099	**341.50**	0.160	0.865

[17] *AUC- area under the ROC curve; p<0.05-signoficative statistic; p<0.001** T2DM-type 2 diabetes mellitus, AF-atrial fibrillation,.*

Table S18. ROC analysis of the NLR, PLR, neutrophils, lymphocytes and platelets in AF+CKD diagnosis

**Diagnostic:** **CKD+AF**	AUC	*p-value*	95% CI L.inf	95% CI L.sup	Gini Index	Cut-off value	Sensitivity	Specificity
Neutrophils / Lymphocyte Ratio	0.646	0.000**	0.609	0.684	0.293	**3.98**	0.607	0.660
Platelet/ Lymphocyte Ratio	0.578	0.000**	0.537	0.620	0.157	**13.76**	0.495	0.644
Neutrophils /ul	0.623	0.000**	0.585	0.661	0.247	**67.15**	0.694	0.535
Lymphocytes /ul	0.349	0.000**	0.312	0.387	-0.301	**4.75**	0.985	0.025
Platelet /ul	0.453	0.037*	0.410	0.497	-0.093	**509.50**	0.031	0.988

[18] *AUC- area under the ROC curv; p<0.05-signoficative statistic; p<0.001** - high significative statistic. CKD-chronic kidney disease, AF-atrial fibrillation*

Table S19. ROC analysis of the NLR, PLR, neutrophils, lymphocytes and platelets in AF+T2DM+CKD diagnosis

**Diagnostic:** **T2DM + CKD + AF**	AUC	*p-value*	95% CI L.inf	95% CI L.sup	Gini Index	Cut-off value	Sensitivity	Specificity
Neutrophils / Lymphocyte Ratio	0.722	0.000**	0.626	0.817	0.444	**5.33**	0.636	0.769
Platelet / Lymphocyte Ratio	0.595	0.114	0.477	0.713	0.190	**23.62**	0.364	0.843
Neutrophils /ul	0.689	0.000**	0.588	0.790	0.379	**74.95**	0.636	0.740

Lymphocytes /ul	0.272	0.000**	0.178	0.366	-0.456	**5.05**	1.000	0.030
Platelet /ul	0.347	0.011*	0.230	0.465	-0.305	**93.50**	1.000	0.024

[19] *AUC- area under the ROC curve; p<0.05-signoficative statistic; p<0.001** - high significative statistic. . T2DM-type 2 diabetes mellitus; CKD-chronic kidney disease, AF-atrial fibrillation*
